# Effects of a Particular Heptapeptide on the IFN-*α*-Sensitive CML Cells

**DOI:** 10.1155/2015/325026

**Published:** 2015-09-01

**Authors:** Fu-lan Yang, Fang-zhi Chen, Xin-xing Wan, Xi Zhou, Mei-juan Zhou, Han-chun Chen, Jun-jiang Fu, Dian-zheng Zhang

**Affiliations:** ^1^Department of Biochemistry, School of Life Sciences and The State Key Laboratory of Medical Genetics, Central South University, Changsha, Hunan 410013, China; ^2^Department of Urology, The Second Xiangya Hospital of Central South University, Changsha, Hunan 410011, China; ^3^Key Laboratory of Epigenetics and Oncology, The Research Center for Preclinical Medicine, Sichuan Medical University, Luzhou, Sichuan 646000, China; ^4^Department of Biochemistry/Molecular Biology, Philadelphia College of Osteopathic Medicine, Philadelphia, PA 19131, USA

## Abstract

Using the phage display biopanning technique, we have previously identified a heptapeptide KLWVIPQ which specifically binds to the surface of the IFN-*α*-sensitive but not the IFN-*α*-resistant CML cells. The effects of this heptapeptide on the IFN-*α*-sensitive CML cells were investigated in the present study. IFN-*α*-sensitive KT-1/A3 and IFN-*α*-resistant KT-1/A3R CML cells were transfected by pEGFP-KLWVIPQ expression vector and/or induced by IFN-*α*. WST-1 cell proliferation assay, flow cytometry, and western blotting were performed to determine the effects of this heptapeptide and/or IFN-*α* on CML cells. The viability of the KT-1/A3 cells was inhibited and apoptosis was induced by either expression of the heptapeptide KLWVIPQ or IFN-*α* treatment with concurrent upregulation of P53 and downregulation of P210^bcr/abl^. However, these effects were not observed in the IFN-*α*-resistant KT-1/A3R cells. These results suggest that the heptapeptide KLWVIPQ shares a similar mechanism with IFN-*α* in the regulation of CML cell growth and apoptosis, implying that the heptapeptide KLWVIPQ could be a novel target to go further into mechanisms of IFN-*α* sensitivity and/or resistance in CML.

## 1. Introduction

Chronic myelogenous leukemia (CML) is a clonal disorder of the multiple pluripotent hematopoietic stem cells characterized by Philadelphia (Ph′) chromosome, which results from a reciprocal chromosomal translocation [t(9;22)(q34;q11)], in which the* bcr* gene on chromosome 22 is fused to the* c-abl* gene on chromosome 9, thereby creating a* bcr-abl* fusion gene [1, 2]. The* bcr-abl* fusion gene encodes a 210-kDa hybrid protein known as P210^bcr/abl^, which has strong tyrosine kinase activity, and is deemed to play a critical role in tumorigenesis of CML [3, 4]. The tyrosine kinase activity of fusion protein P210^bcr/abl^ leads to uncontrolled cell proliferation with suppressed apoptosis and then results in the malignant expansion of multipotential hematopoietic stem cells in bone marrow [5]. P210^bcr/abl^ protein activates multiple signal transduction pathways such as phosphatidylinositol 3 kinase, Ras/Raf/mitogen-activated protein kinase (MAPK), and STAT5/Janus kinase pathways to achieve its functions [6, 7].

Interferon-alpha (IFN-*α*) has been used for decades to treat viral malignancy because of its broad range of biologic activities, including mediation of the beneficial immunomodulatory effects [8, 9], suppression of malignant cell proliferation, and inducement of apoptosis [10]. Based on inducing hematologic and cytogenetic remissions in many patients with CML in chronic phase, IFN-*α* has been extensively used for CML patients treatment in an era [11], but it fails to effectively induce long-term cytogenetic remission in some CML patients [12]. As one of the most effective pharmaceuticals for CML treatment over the past two decades, the mechanisms of IFN-*α* treatment are not fully understood. IFN-*α* can elicit multiple biologic functions because of its diverse signaling pathways, including Rap1, CrkL, VAV, MAP kinase, and PI3-kinase [13–15]. Although IFN-*α* is effective in achieving control of CML in most patients, the resistance of CML to IFN-*α* might emerge* de novo* or during treatment and ultimately leads to disease progression [16]. There are many effective targeting-pharmaceuticals such as tyrosine kinase inhibitor imatinib [17, 18] which leads to the study and use of IFN-*α* dramatically reduced in the last decade [19]. It is evident that any malignancy results from multiple pathogenic factors, and the related studies showed that any anticancer molecule was not universally effective to tumors [20]. IFN-*α*, which is associated with a broad range of therapeutic effects and influences multiple events, might be more valuable for use in combination with the other targeting agents to effectively treat leukemia patients than use alone [21]. In a study by Palandri et al. [22], it was suggested that the response to the combination of imatinib with IFN-*α* is more rapid and effective than imatinib alone for treatment of CML in chronic phase. El Eit et al. [23] found that combined effects between arsenic and IFN treatment,* in vivo*, can prolong the survival of primary CML mice. Some recent findings suggested that combination therapy of IFN-*α* with imatinib to CML increases the speed and rate of responses [24, 25]. Katagiri et al. [26] observed that treatment-free molecular remission achieved by combination therapy of imatinib plus IFN-*α* in CML with BCL2L11 (BIM) deletion polymorphism relapsed after stopping imatinib. So, the resistance of CML to the used therapeutics or combination treatment is still an observable fact. The further studies for the mechanisms of IFN-*α* action are needed to clarify the best niche for IFN-*α* use in CML.

In an early study, our research group identified some heptapeptides that can specifically bind to the surface of IFN-*α*-sensitive CML cell line KT-1/A3 cells using phage display library. One of the heptapeptides, KLWVIPQ, has a partial amino acid sequence homology with the C-terminal domain of E3 ubiquitin-protein ligase (E3) [27]. In the present study, this heptapeptide KLWVIPQ has been chosen to treat IFN-*α*-sensitive KT-1/A3 and IFN-*α*-resistant KT-1/A3R cells to explore the regulatory effects of the IFN-*α*-sensitive CML cell-binding heptapeptide KLWVIPQ on proliferation, apoptosis, and the related-gene expression of CML cells. The results would contribute to a novel target to go further into mechanisms of IFN-*α* sensitivity and/or resistance for clearing the best niche of IFN-*α* use in CML.

## 2. Materials and Methods

### 2.1. Construction of the Recombinant Eukaryotic Expression Vector

Based on the amino acids sequence of the heptapeptide KLWVIPQ, a specific DNA fragment with the sequence of AAG CTG TGG GTA ATC CCA CAG was designed. In order to destine the expressed heptapeptide outside of the cell, a DNA fragment encoding the signal peptide (from the* Mus musculus* immunoglobulin heavy chain complex) ATG AAC TTC GGG CTC AGC TTG ATT TTC CTT GTC CTT GTT TTA AAA GGT GTC CAG TGT GAA was added in front of the heptapeptide DNA sequence. For the purpose of PCR and subcloning, extra sequences were added to both ends of the expressing DNA sequence to make the DNA fragment in 159 bp length:  GCT AGC GCT ACC GGA CTC AGA T *C* ^*∗*^ *T CGA G*CT CAA GCT TCG AAT TCT GCA GTC GAC GGT ACC G ATG AAC TTC GGG CTC AGC TTG ATT TTC CTT GTC CTT GTT TTA AAA GGT GTC CAG TGT GAA** AAG CTG TGG GTA ATC CCA CAG** AGT AGT CT *A CCG G* ^*∗*^ *T* C GC. A pair of PCR primers (forward: 5′-GCT AGC GCT ACC GGA CTC AGA TCT-3′, reverse: 5′-GCG ACC GGT AGA CTA CTC TGT G-3′) were designed according to the underlined sequences. Two restriction sites (*Age I* and* Xho I*) were shown in italic. This DNA fragment was synthesized by BGI (China) and cloned into the pEGFP-N1 eukaryotic expression vector (Takara, Japan) between the sites of* Age I* and* Xho I*. The inserted sequence was confirmed by DNA sequencing and the recombinant plasmid was named as pEGFP-KLWVIPQ.

### 2.2. Cell Culture and Transfection

Human CML cell lines KT-1/A3 and KT-1/A3R (kindly provided by Dr. Sakai, Ehime University, Japan) were cultured in RPMI1640 medium (GIBCO, USA) with 10% fetal bovine serum (GIBCO, USA) at 37°C with 5% CO_2_. Cells were transiently transfected with pEGFP-KLWVIPQ and the empty vector pEGFP-N1 by using Sofast transfection reagent (Sunmabio, China). According to the manufacturer's instruction of transfection reagent kit, the plasmid (2 *μ*g) and reagent (5 *μ*L) were prepared, respectively, and transferred to KT-1/A3 and KT-1/A3R cells on 6-well tissue culture plates with 2 mL of 4 × 10^5^/mL cell suspension in each well. The transfected cells were cultured in presence or absence of IFN-*α* (Sigma, USA) in 1000 U/mL. At 48 h after transfection, the cells were washed with phosphate buffered saline (PBS) for testing.

### 2.3. Cell Growth Assays

Cell proliferation was measured by using the WST-1 cell proliferation and cytotoxicity assay kit (Beyotime, China). Cells were cultured in 96-well plates at 3 × 10^5^/mL in a total volume of 100 *μ*L for every clone. After 48 h of incubation, 10 *μ*L WST-1 reagents were added to each well, and the cell proliferation rates were measured by detecting A450 values. Mean and standard deviation were calculated from triplicate experiments. Flow cytometry was employed for checking apoptosis rates of the transfected cells.

### 2.4. Gene Expression Analysis

Expression levels of the interested genes were tested by western blotting. 1 × 10^6^ cells were harvested after stimulation with the expressed heptapeptide KLWVIPQ and/or IFN-*α* for 48 h. After being washed by PBS buffer, the cells were immediately placed on ice and lysed in 100 *μ*L of cold radio immunoprecipitation assay (RIPA) lysis buffer at 4°C for 15 min. The insoluble fraction was removed by centrifugation at 10000 g for 20 min. The protein concentration was measured using Pierce BCA protein assay kit (Thermo, USA). 30 *μ*g of proteins for each lane was loaded onto 10% SDS-polyacrylamide gel. Following electrophoresis, the proteins were transferred onto polyvinylidene fluoride (PVDF) membrane. The PVDF membrane was blocked by incubation in Tris-buffered saline containing 5% nonfat dry milk and 0.1% Tween 20 for 1 h at room temperature and then incubated at 4°C overnight with the antibody of mouse anti-human P53 or P210^bcr/abl^ (Cell Signalling Technology Inc., USA). The PVDF membrane was washed three times in the Tris-buffered saline containing 0.1% Tween 20 and then incubated with the enhanced chemiluminescence labeled-antibody against mouse P53 antibody or P210^bcr/abl^ antibody (Beyotime, China) for 1 h. The PVDF membrane was washed again as described above and the bond antibodies were detected by using the enhanced chemiluminescence kit (Beyotime, China).

### 2.5. Statistical Analysis

Experiments were repeated at least three times and the data were calculated as mean ± standard deviation. The data were analyzed using SPSS 16.0 software. Differences were analyzed using the paired-samples *t*-test and the one-way analysis of variance (ANOVA) to compare the differences between groups.

## 3. Results

### 3.1. Construction and Transfection of Recombinant Expression Vector pEGFP-KLWVIPQ

**Figure 1:**
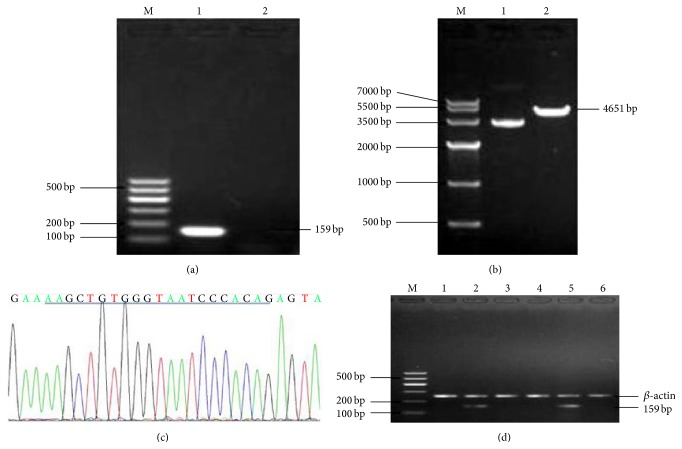
Construction and characterization of the plasmid expressing KLWVIPQ. (a) Agarose gel analysis of the PCR products amplified from positive (lane 1) and negative (lane 2) plasmids. Lane M: DNA molecular weight marker. (b) Agarose gel electrophoresis of the pEGFP-KLWVIPQ plasmid without (lane 1) and with (lane 2) the digestions of* XhoI* and* AgeI*. (c) Sequencing results of the recombinant plasmid pEGFP-KLWVIPQ. (d) Agarose gel analysis of the PCR products amplified from the DNA purified from KT-1/A3 (lanes 1–3) and KT-1/A3R (lanes 4–6) cells under different transfection conditions. Lanes 1 and 4: without transfection, lanes 2 and 5: transfected with pEGFP-KLWVIPQ, and lanes 3 and 6: transfected with the empty vector pEGFP-N1.

The DNA fragment encoding the heptapeptide KLWVIPQ with a signal peptide sequence was inserted between the* XhoI* and* AgeI* restriction sites in the expression vector pEGFP-N1. To verify the insertion of the designed DNA fragment, PCR was conducted with specific primers using the plasmid DNA as template. As shown in Figure 1(a), a fragment of DNA with expected size of 159 bp was successfully amplified with the positive plasmid (lane 1), but PCR with the empty vector showed no amplification (lane 2).  Figure 1(b) showed the positive plasmid before (lane 1) and after (lane 2) the digestion with* XhoI* and* AgeI*. The results from DNA sequencing (Figure 1(c)) demonstrated that the specific DNA sequence was successfully inserted into the plasmid vector as designed. To confirm that the recombinant plasmid pEGFP-KLWVIPQ was transfected into the KT-1/A3 and KT-1/A3R cells, RT-PCR was conducted. As shown in Figure 1(d), the expected 159 bp fragments were only amplified in the cells transfected with the positive clone (lanes 2 and 5) and not amplified in the cells either without transfection (lanes 1 and 4) or transfected with the empty vector (lanes 3 and 6). Altogether, these results demonstrated that we have not only constructed the plasmid containing the DNA encoding the heptapeptide, but also successfully transfected it into the KT-1/A3 and KT-1/A3R cells.

### 3.2. The Effects of KLWVIPQ and IFN-*α* on the Viability of KT-1/A3 and KT-1/A3R Cells

**Figure 2:**
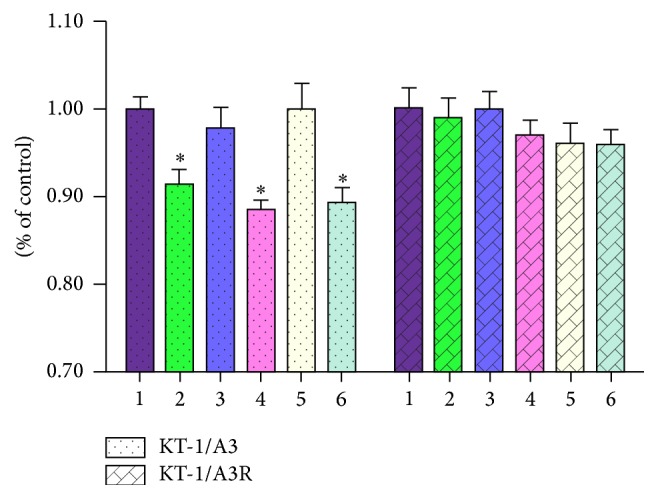
The effects of KLWVIPQ expression and IFN-*α* on KT-1/A3 and KT-1/A3R cell viability (*n* = 3). The KT-1/A3 and KT-1/A3R cells were transfected or treated with IFN-*α* and viable cells were estimated. 1: control; 2: transfection with pEGFP-KLWVIPQ; 3: transfection with pEGFP-N1; 4: IFN-*α* treatment; 5: transfection with pEGFP-KLWVIPQ and IFN-*α* treatment; 6: transfection with pEGFP-N1 and IFN-*α* inducement. ^*∗*^Significant difference between test and control groups.

**Figure 3:**
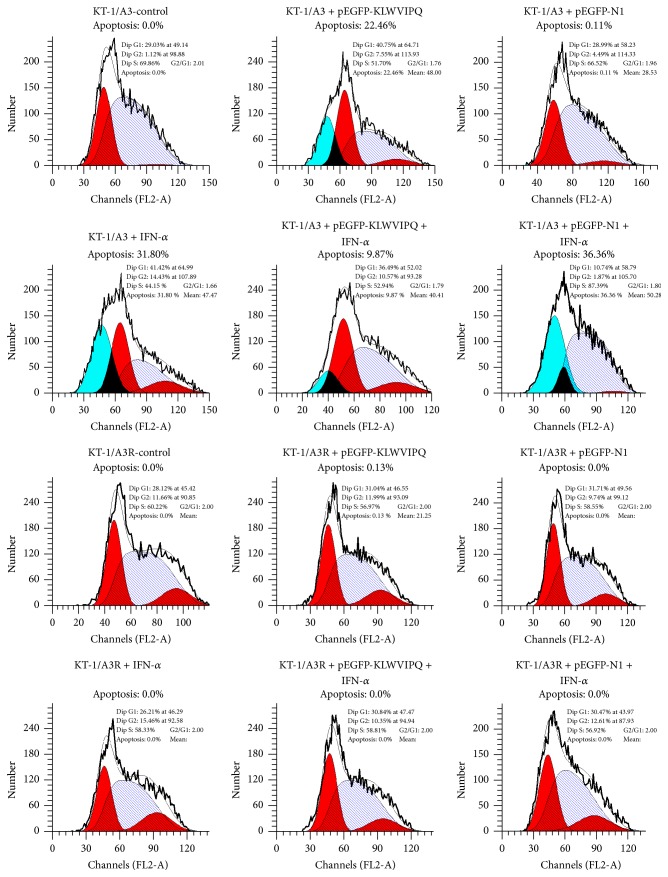
The effects of expressing KLWVIPQ or IFN-*α* treatment on KT-1/A3 and KT-1/A3R cell apoptosis. The KT-1/A3 and KT-1/A3R cells were either pEGFP-KLWVIPQ transfection or IFN-*α* treatment alone, or in combination, and analyzed by flow cytometry. Nontreatment or transfection with the empty vector served as negative controls. The apoptotic populations were indicated as percentages.

The cells were either transfected with the expressing plasmid or treated with IFN-*α* alone or in combination and the number of viable cells was estimated. As shown in Figure 2, the viability of KT-1/A3 cells was significantly reduced by either expressing the heptapeptide or IFN-*α* inducement comparing to the nontreatment control (*n* = 3, *P* < 0.05). Transfection of the empty vector has no effect on the cell viability. However, the repression effects of heptapeptide and IFN-*α* on cell viability canceled with each other when the cells were transfected with expression vector together with IFN-*α* treatment. Nevertheless, the above observed effects on the KT-1/A3 cells were not seen on the KT-1/A3R cells. Then, we analyzed the apoptotic status of the cells under different treatments by flow cytometry. As shown in Figure 3, transfection of the empty vector has no effect on the apoptotic status of the cells. Transfection of the pEGFP-KLWVIPQ or treatment with IFN-*α* alone increased the apoptotic cell population by 22.46% and 31.80%, respectively. However, the apoptotic cell population reduced to 9.87% when the cells were transfected with the pEGFP-KLWVIPQ and treated with IFN-*α* together. Similarly, as observed in the cell viability, these treatments to the KT-1/A3R cells showed no effect on the apoptotic population.

### 3.3. The Effects of pEGFP-KLWVIPQ Transfection and IFN-*α* Treatment on Gene Expression

**Figure 4:**
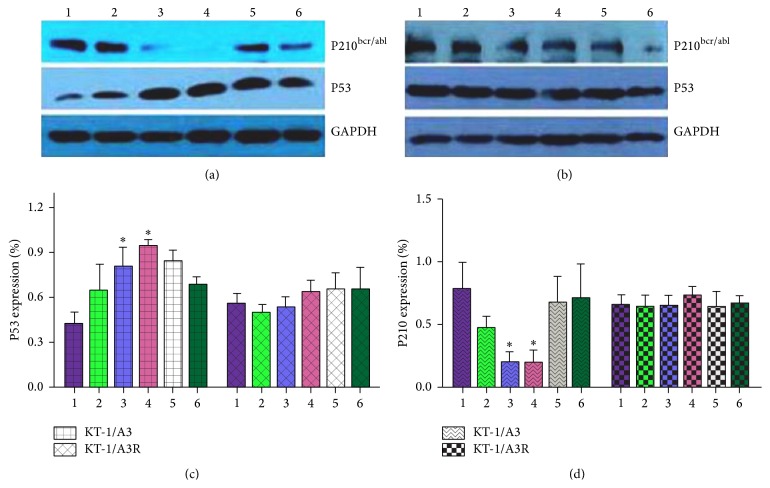
The levels of P53 and P210^bcr/abl^ in KT-1/A3 and KT-1/A3R cells when transfected with pEGFP-KLWVIPQ and treated with IFN-*α* (*n* = 3). KT-1/A3 (a) and KT-1/A3R (b) cells were either transfected with pEGFP-KLWVIPQ or treated with IFN-*α* alone, or in combination, and the cell lysates were analyzed by western blot assays with antibodies against P53, P210^bcr/abl^, and GAPDH. The intensities of the bands were semiquantified and ratios of P53/GAPDH and P210^bcr/abl^/GAPDH were plotted (c and d). The values showed were means ± standard deviation of triplicate experiments (^*∗*^ *P* < 0.05). 1: control; 2: 1000 U/mL of IFN-*α*; 3: 3000 U/mL of IFN-*α*; 4: expression of KLWVIPQ; 5: expression of KLWVIPQ with 1000 U/mL of IFN-*α*; 6: expression of KLWVIPQ with 3000 U/mL of IFN-*α*. ^*∗*^Significant difference between the test and control groups.

The tumor suppressor P53 that functions to initiate cell cycle arrest and apoptotic pathways in cells has been found to be upregulated in response to IFN-*α* [9, 28]. P210^bcr/abl^ is characterized as the specific biomarker of CML and its expression level symbolizes the progression of CML [29]. We were interested in understanding if the effects of the heptapeptide KLWVIPQ on KT-1/A3 cells were also P53- and P210^bcr/abl^-dependent. As shown in Figure 4(a), P210^bcr/abl^ and P53 were down- and upregulated by IFN-*α* in a dose-dependent manner comparing to the no-treatment control. Transfection of the pEGFP-KLWVIPQ to KT-1/A3 cell also down- and upregulated P210^bcr/abl^ and P53, respectively. However, expression of the peptide KLWVIPQ counteracted the IFN-*α* effect on these genes. These effects were reproducible and statistically significant (Figure 4(c)). However, when the KT-1/A3R cells underwent the same treatments, the levels of P53 and P210^bcr/abl^ were affected minimally (Figures 4(b), 4(c), and 4(d)).

## 4. Discussion

The heptapeptide KLWVIPQ was previously identified during the screening of a phage display peptide library [27]. This peptide specifically binds to the surface of the IFN-*α*-sensitive KT-1/A3 but not that of the IFN-*α*-resistant KT-1/A3R CML cells. Further sequence analyses found that this heptapeptide shares a partial sequence (LWVIP) homology with the C-terminal domain of the E3 ubiquitin-protein ligase. E3 ligase is responsible for specific protein ubiquitination and subsequent degradation and therefore E3 ligase plays an important role in controlling the levels of various cellular proteins [30]. Ubiquitination also serves as one of the major signaling pathways in determining apoptosis [31]. In this study, we expressed this heptapeptide KLWVIPQ by transfecting the expressing plasmid into the IFN-*α*-sensitive KT-1/A3 and the IFN-*α*-resistant KT-1/A3R CML cell lines to decipher the roles of the heptapeptide KLWVIPQ in IFN-*α*-mediated effects on CML. Expressing this heptapeptide inhibited KT-1/A3 cell growth and induced apoptosis. However, expression of this peptide counteracted the IFN-*α*-regulated gene expression and KT-1/A3 cell apoptosis. Given the partial homology between this heptapeptide and E3 ligase, we postulated that this particular heptapeptide may alternate the IFN-*α* regulated P53 and P210^bcr/abl^ expression by interfering with E3 ligase-mediated protein ubiquitination/degradation pathway.

In order to avoid the detrimental effects from overreaction to type I IFN, a timely downregulation of the response to type I IFN signal is needed and the HECT domain of ubiquitin E3 ligase RAUL is essential in this process. Zheng et al. [32] suggested that IFN-*α* limits the extent of cellular responses to IFN-*α* by inducing the PKD2, and the activated PKD2 subsequently facilitates ubiquitination-mediated IFNAR1 degradation through interacting with E3 ubiquitin ligase *β*Trcp. RAUL limits type I IFN production by directly catalyzing lysine 48-linked poly-ubiquitination of both interferon regulatory factor 7 (IRF7) and IRF3 followed by proteasome-dependent protein degradation [33]. In addition, both ubiquitin E3 ligase Efp and interferon stimulated gene 15 (ISG15) were upregulated by IFN in HepG2 and HeLa cells [34, 35]. In order to investigate the regulatory effects of the IFN-*α*-sensitive CML cell-binding heptapeptide KLWVIPQ partially homologized with E3 ligase on the related protein levels and cell growth of CML cells, a novel recombinant expression plasmid expressing secretive heptapeptide KLWVIPQ was established in the present study. After pEGFP-KLWVIPQ transfection in the condition with or without IFN-*α* inducement, the status of KT-1/A3 and KT-1/A3R CML cells in proliferation and apoptosis and the related protein levels were evaluated by using cell and molecular technologies.

WST-1 cell proliferation analysis showed that proliferation of KT-1/A3 cells was suppressed when the cells were independently treated by pEGFP-KLWVIPQ transfection or IFN-*α* inducement. However, the suppression effects were reduced when the cells were treated by pEGFP-KLWVIPQ and IFN-*α* together. Tingle-trial flow cytometry results indicate that either pEGFP-KLWVIPQ expression or IFN-*α* treatment can stimulate apoptosis of KT-1/A3 cells. However, like the inhibitory effects of pEGFP-KLWVIPQ and IFN-*α* on growth of KT-1/A3 cells, the effects of pEGFP-KLWVIPQ and IFN-*α* on apoptosis of KT-1/A3 cells were reduced when they were used together. The reasons underlying such a phenomenon are obscure. The KT-1/A3R cells kept no response to the expressed peptide KLWVIPQ and/or IFN-*α*.

P53 is a key regulator of apoptosis and is also upregulated by IFN [36]. The tyrosine kinase protein P210^bcr/abl^ as the specific biomarker of CML is encoded by the* bcr-abl* fusion gene. Both P53 and P210^bcr/abl^ play critical roles in the pathogenesis and progression of CML [4]. Additionally, inhibition of* bcr-abl* fusion gene expression has also been suggested as one of the mechanisms involved in IFN-*α* function for CML treatment [37]. We found that IFN-*α* was able to upregulate P53 and downregulate P210^bcr/abl^ in the IFN-*α*-sensitive KT-1/A3 cells, but not the IFN-*α*-resistant KT-1/A3R cells. These results suggest that lost regulation of P53 and P210^bcr/abl^ could be one of the explanations for the CML resistance to IFN-*α* treatment. Interestingly, the heptapeptide KLWVIPQ showed similar effects on the expression of P53 and P210^bcr/abl^ in the IFN-*α*-sensitive KT-1/A3 cells, but not the IFN-*α*-resistant KT-1/A3R cells. Given the fact that this particular heptapeptide KLWVIPQ binds to the IFN-*α*-sensitive KT-1/A3 cells, it is likely that certain molecules responsible for the interaction with this particular heptapeptide are specifically expressed on the surface of the IFN-*α*-sensitive KT-1/A3 cells. On the other hand, the IFN-*α*-resistant KT-1/A3R cells somehow managed to downregulate the expression of these molecules which render the cells to escape the IFN-*α*-mediated cell death and therefore become IFN-*α*-resistant. If this proved to be true, identification and upregulation of this molecule could become a valuable strategy in turning the CML cells from IFN-*α*-resistant to IFN-*α*-sensitive. In addition, since overexpression of the heptapeptide KLWVIPQ counteracted IFN-*α*-mediated regulation of P53 and P210^bcr/abl^ as well as KT-1/A3 cell apoptosis, we speculate that this heptapeptide and IFN-*α* compete with the same or similar cell surface binding site on the IFN-*α*-sensitive KT-1/A3 cells. Therefore, either this heptapeptide or IFN-*α* could be used as a bait in fishing the molecule specifically expressed on the surface of the IFN-*α*-sensitive KT-1/A3 cells.

In summary, these results collectively demonstrated that the heptapeptide KLWVIPQ was capable of suppressing proliferation, accelerating apoptosis, and up- and downregulating P53 and P210^bcr/abl^ expression, respectively, for the IFN-*α*-sensitive KT-1/A3 CML cells but has no effects on IFN-*α*-resistant KT-1/A3R CML cells. Strategies inducing the expression of the specific molecule responsible for the binding of this peptide on the surface of IFN-*α*-resistant cells could make the cells become IFN-*α*-sensitive. Finally, this particular heptapeptide could be a novel target to go further into mechanisms of IFN-*α* action in CML.
